# Supplementary material for: Limited Marginal Utility of Deep Sequencing for HIV Drug Resistance Testing in the Age of Integrase Inhibitors
Source: J Clin Microbiol. 2018 Nov 27;56(12):e01443-18. doi: 10.1128/JCM.01443-18 (PMC6258839; doi:10.1128/JCM.01443-18)
Supplement: Supplemental file 1 [file zjm012186202s1.pdf]

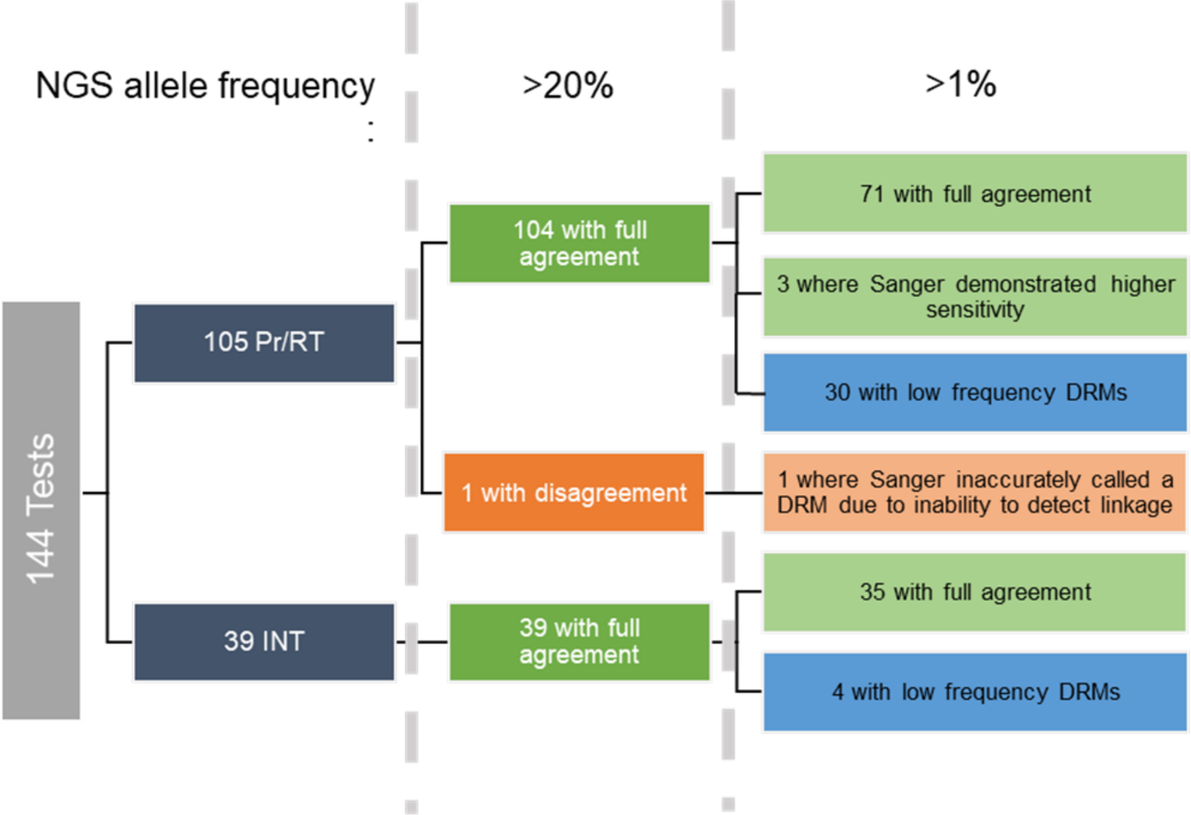

Figure S-1. Agreement of Sanger and NGS variant resistance interpretations at 20% and 1% cutoffs. Low frequency DRMs refer to those between 1-20% frequency and were only called by NGS. Tests with agreement between Sanger and NGS (green); disagreement due to low frequency DRMs (blue); Sanger false-positive due to inability to detect linkage (orange).
