# Supplementary material for: Limited Marginal Utility of Deep Sequencing for HIV Drug Resistance Testing in the Age of Integrase Inhibitors
Source: J Clin Microbiol. 2018 Nov 27;56(12):e01443-18. doi: 10.1128/JCM.01443-18 (PMC6258839; doi:10.1128/JCM.01443-18)
Supplement: Supplemental file 2 [file zjm012186202s2.pdf]

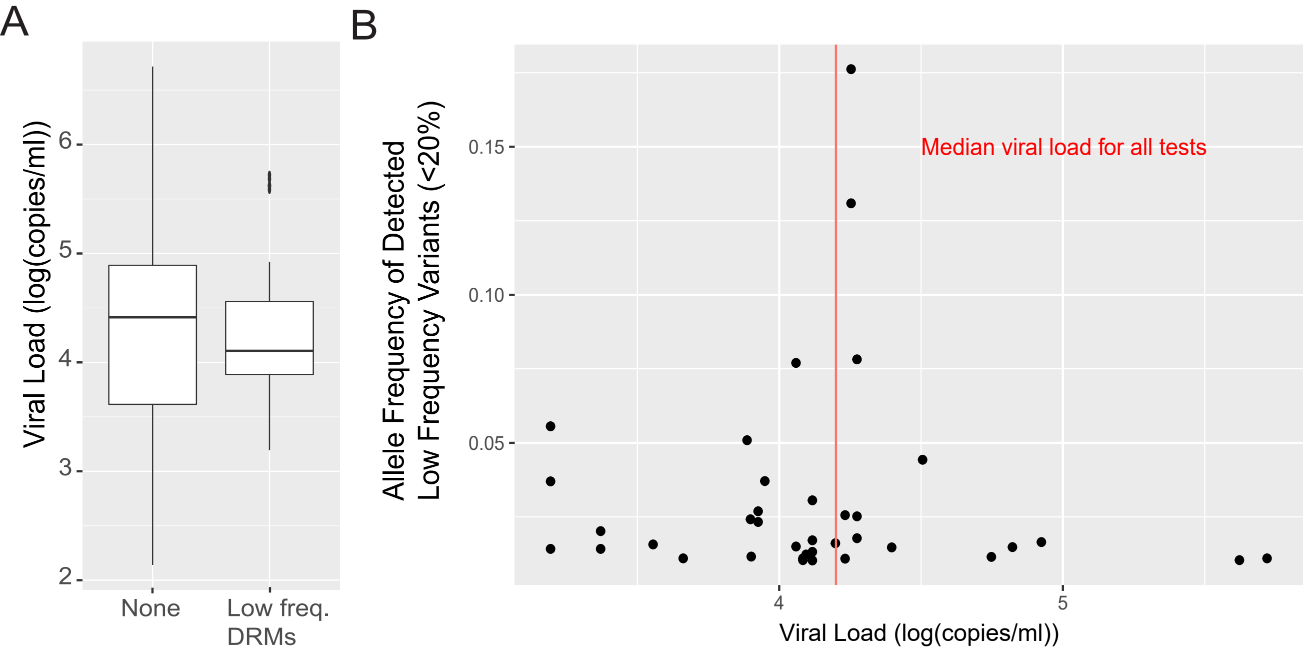

Figure S-2. Low frequency variants detected in patients with a range of viral loads.
A) Boxplot distribution of patients' viral load [plasma HIV RNA log(copies/mL)] at sample date for samples with and without detected low frequency DRMs. B) Viral load of patient in which a low frequency DRM was detected and the allele frequency of the detected DRM(s).
