# Supplementary material for: Limited Marginal Utility of Deep Sequencing for HIV Drug Resistance Testing in the Age of Integrase Inhibitors
Source: J Clin Microbiol. 2018 Nov 27;56(12):e01443-18. doi: 10.1128/JCM.01443-18 (PMC6258839; doi:10.1128/JCM.01443-18)
Supplement: Supplemental file 3 [file zjm012186202s3.pdf]

Table S-1. Primers used in nested PCR amplification of Pr/RT and INT.

Gene	Primer Name	Primer	Direction	HXB2 Numbering
Pr/RT	NEF10	GARAGACAGGCTAATTTTTTAGGGA	Forward	2071-2095
	245.RT	TACTTTAATTCCTGSRTAAATCTGACTTG	Reverse	3349-3377
	NEF11	CAAATCACTCTTTGGCARCGACC	Forward	2256-2278
	NER10	AAYTTCTGTATATCATTGACAGTCCA	Reverse	3328-3303
INT	INT-FO	AGTAAACATAGTAACAGACTCACARTATG	Forward	4025-4053
	1.3'INT	CCTAGTGGGATGTGTACTTCTGAACTTA	Reverse	5193-5220
	2.5'INT	GTAGCAAAAGAAATAGTAGCCAGCTGTGATAAAT	Forward	4324-4357
	2.3'INT	GTTTTACTAAACTTTTCCATGTTCTAATCCTCAT	Reverse	5087-5120

Table S-2. Complete set of 105 PR/RT and 39 IN NGS and Sanger sequences submitted to GenBank.

Sample ID (HIV-#)	Gene segment	NGS Accession #	Sanger ID	Genbank Accession #
1	INT	SRX4580764	FHS68	MH789585
2	Pr/RT	SRX4580765	FHS52	MH789576
2	INT	SRX4580765	FHS69	MH789586
3	Pr/RT	SRX4580766	FHS53	MH789577
3	INT	SRX4580766	FHS70	MH789587
4	Pr/RT	SRX4580767	FHS54	MH789579
5	Pr/RT	SRX4580760	FHS55	MH789578
6	Pr/RT	SRX4580761	FHS56	MH789575
7	Pr/RT	SRX4580762	FHS57	MH789580
9	Pr/RT	SRX4580763	FHS59	MH789582
10	Pr/RT	SRX4580768	FHS60	MH789581
11	Pr/RT	SRX4580769	FHS61	MH789583
14	Pr/RT	SRX4580789	FHS65	MH789584
21	Pr/RT	SRX4580790	FHT53	MH789588
22	Pr/RT	SRX4580787	FHT54	MH789589
23	Pr/RT	SRX4580788	FHT56	MH789591
23	INT	SRX4580788	FHT71	MH789599
25	Pr/RT	SRX4580785	FHT59	MH789594
25	INT	SRX4580785	FHT72	MH789598
26	Pr/RT	SRX4580786	FHT60	MH789592
27	Pr/RT	SRX4580783	FHT61	MH789593
27	INT	SRX4580783	FHT73	MH789600
28	Pr/RT	SRX4580784	FHT62	MH789595
29	Pr/RT	SRX4580791	FHT64	MH789596
29	INT	SRX4580791	FHT76	MH789601
30	Pr/RT	SRX4580792	FHT65	MH789597
33	INT	SRX4580806	FHT80	MH789602
34	Pr/RT	SRX4580805	FHU51	MH789603
36	Pr/RT	SRX4580804	FHU54	MH789605

40	Pr/RT	SRX4580803	FHU59	MH789604
42	Pr/RT	SRX4580810	FHU61	MH789608
43	INT	SRX4627405	FHU77	MH838015
45	Pr/RT	SRX4580808	FHU63	MH838017
46	Pr/RT	SRX4580807	FHU64	MH789606
47	INT	SRX4580812	FHU79	MH838014
47	Pr/RT	SRX4580812	FHU65	MH789607
48	Pr/RT	SRX4580811	FHU66	MH789609
49	Pr/RT	SRX4627406	FHU69	MH789610
50	Pr/RT	SRX4580819	FHW52	MH789612
52	Pr/RT	SRX4580820	FHW54	MH789613
53	Pr/RT	SRX4580821	FHW55	MH789614
54	Pr/RT	SRX4580822	FHW56	MH789615
56	Pr/RT	SRX4580815	FHW59	MH789616
58	Pr/RT	SRX4580816	FHW61	MH789617
59	Pr/RT	SRX4580817	FHW62	MH789618
59	INT	SRX4580817	FHW78	MH789621
60	Pr/RT	SRX4580818	FHW64	MH789620
61	Pr/RT	SRX4580813	FHW68	MH789619
64	Pr/RT	SRX4580814	FHX155	MH789625
65	Pr/RT	SRX4580826	FHX151	MH789622
67	Pr/RT	SRX4580825	FHX153	MH789623
68	Pr/RT	SRX4580828	FIA51	MH789629
69	Pr/RT	SRX4580827	FIA53	MH789630
69	INT	SRX4580827	FIA73	MH789636
70	Pr/RT	SRX4580830	FIA54	MH789631
70	INT	SRX4580830	FIA74	MH789639
71	Pr/RT	SRX4580829	FIA55	MH789632
72	Pr/RT	SRX4580832	FIA56	MH789633
73	Pr/RT	SRX4580831	FIA57	MH789634
75	Pr/RT	SRX4580824	FIA61	MH789635
75	INT	SRX4580824	FIA78	MH789640
79	Pr/RT	SRX4580823	FHZ51	MH789624
80	Pr/RT	SRX4580842	FIA65	MH789637
82	Pr/RT	SRX4580843	FHZ69	MH789628
85	INT	SRX4580840	FIA77	MH789638
86	INT	SRX4580841	FIA79	MH838016
88	Pr/RT	SRX4580846	FIB152	MH789642
89	Pr/RT	SRX4580847	FIB153	MH789643
90	Pr/RT	SRX4580844	FIB154	MH789646
95	Pr/RT	SRX4580845	FIB60	MH789641
96	Pr/RT	SRX4580837	FIB155	MH789644
98	Pr/RT	SRX4580838	FHZ56	MH789626
100	Pr/RT	SRX4580870	FIC51	MH789645
101	Pr/RT	SRX4580869	FIC53	MH789647

102	Pr/RT	SRX4580868	FIC54	MH789648
102	INT	SRX4580868	FIC70	MH789652
104	Pr/RT	SRX4580867	FIC56	MH789649
104	INT	SRX4580867	FIC71	MH789654
107	Pr/RT	SRX4580866	FIC59	MH789651
108	INT	SRX4580865	FIC75	MH838013
108	Pr/RT	SRX4580865	FIC61	MH789650
113	INT	SRX4580864	FIC74	MH789653
117	Pr/RT	SRX4580863	FID53	MH789655
119	Pr/RT	SRX4580872	FID55	MH789656
119	INT	SRX4580872	FID67	MH789658
122	Pr/RT	SRX4580871	FID61	MH789657
125	Pr/RT	SRX4580873	FIE51	MH789659
125	INT	SRX4580873	FIE71	MH789667
126	Pr/RT	SRX4580874	FIE52	MH789660
126	INT	SRX4580874	FIE72	MH789668
127	Pr/RT	SRX4580875	FIE53	MH789661
127	INT	SRX4580875	FIE73	MH789669
128	Pr/RT	SRX4580876	FIE54	MH789662
128	INT	SRX4580876	FIE74	MH789670
130	Pr/RT	SRX4580877	FIE58	MH789664
131	Pr/RT	SRX4580878	FIE59	MH789663
132	Pr/RT	SRX4580879	FIE60	MH789665
133	Pr/RT	SRX4580880	FIE61	MH789666
133	INT	SRX4580880	FIE77	MH789671
134	INT	SRX4580881	FIE79	MH789672
140	INT	SRX4580882	FIE83	MH789673
145	Pr/RT	SRX4580850	FIF55	MH789675
146	Pr/RT	SRX4580849	FIF56	MH789674
146	INT	SRX4580849	FIF67	MH789677
148	Pr/RT	SRX4580852	FIF58	MH789676
150	Pr/RT	SRX4580851	FIG52	MH789678
151	Pr/RT	SRX4580839	FIG53	MH789679
152	Pr/RT	SRX4580836	FIG54	MH789682
153	Pr/RT	SRX4580848	FIG55	MH789680
154	Pr/RT	SRX4580770	FIG57	MH789681
155	INT	SRX4580772	FIG61	MH789683
156	INT	SRX4580771	FIG62	MH789684
157	INT	SRX4580801	FIG64	MH789685
160	Pr/RT	SRX4580802	FIH53	MH789686
161	Pr/RT	SRX4580799	FIH54	MH789687
163	Pr/RT	SRX4580800	FIH56	MH789688
164	Pr/RT	SRX4580797	FIH58	MH789689
165	Pr/RT	SRX4580798	FIH59	MH789690
166	INT	SRX4580795	FII64	MH789700

168	INT	SRX4580793	FIH67	MH838012
169	INT	SRX4580794	FIH68	MH789691
170	INT	SRX4580782	FIH70	MH789692
171	Pr/RT	SRX4580781	FII52	MH789694
173	Pr/RT	SRX4580780	FII54	MH789693
174	Pr/RT	SRX4580779	FII55	MH789696
175	Pr/RT	SRX4580778	FII56	MH789695
176	Pr/RT	SRX4580777	FII57	MH789697
177	Pr/RT	SRX4580776	FII58	MH789698
178	Pr/RT	SRX4580775	FII61	MH789699
179	Pr/RT	SRX4580774	FII64	MH789700
180	Pr/RT	SRX4580773	FII65	MH789701
181	Pr/RT	SRX4580853	FII66	MH789703
182	INT	SRX4580854	FII69	MH838011
183	INT	SRX4580855	FII73	MH789702
184	INT	SRX4580856	FII77	MH789704
185	Pr/RT	SRX4580857	FIJ51	MH789706
186	Pr/RT	SRX4580858	FIJ52	MH789705
188	Pr/RT	SRX4580859	FIJ55	MH789707
189	Pr/RT	SRX4580860	FIJ56	MH789708
190	Pr/RT	SRX4580861	FIJ57	MH789709
191	Pr/RT	SRX4580862	FIJ58	MH789710
192	Pr/RT	SRX4580834	FIJ59	MH789712
193	Pr/RT	SRX4580833	FIJ60	MH789711
194	Pr/RT	SRX4580835	FIJ64	MH789713
